# Describing the validity of carcinogen screening tests.

**DOI:** 10.1038/bjc.1979.10

**Published:** 1979-01

**Authors:** J. A. Cooper, R. Saracci, P. Cole


*Br. J. Cancer* (1979) **39**, 87

***Short Communication***

### DESCRIBING THE VALIDITY OF CARCINOGEN SCREENING TESTS

J. A. COOPER II*, R. SARACCI† AND P. COLE†

*From the *Division of Cancer Cause and Prevention, National Cancer Institute, Bethesda, Maryland,* 20014, *USA and the †Unit of Epidemiology and Biostatistics, International Agency for Research on Cancer,* 69372 *Lyon Cédex* 2, *France*

Received 28 August 1978   Accepted 16 October 1978

SEVERAL issues pertinent to the validation of screening tests and to the evaluation of screening programmes have evolved, largely in the epidemiological and general medical literature (Vecchio, 1966; Holland, 1974; Henderson, 1976). For this reason, laboratory scientists interested in the development and validation of carcinogen screening tests may not be fully aware of some useful concepts. The paper reviews several such concepts and their relationships. These relationships explain, for example, why the "predictive value", although frequently used as an index of the utility of a screening test, is not well suited for that purpose.

A carcinogen screening test is usually evaluated by applying it to a group of substances, each of which is considered to be, or not to be, a carcinogen according to some selected criteria (*i.e.* animal or human carcinogenesis). The specific criteria chosen are beyond the scope of this discussion; however, the validity measures of a screening test are meaningful only in the context of these criteria. After some number, N, of substances has been tested, the results may be described using the format of the Table. (Terms used to describe screening tests and screening programmes are defined below the Table.)

A screening test is expected to designate carcinogens as "positive" and to designate non-carcinogens as "negative". Each of these 2 distinct functions has its own measure of validity. The proportion of carcinogens which give a positive result in the test is termed the sensitivity. The proportion of non-carcinogens which give a negative result is termed the specificity (MacMahon & Pugh, 1970). The sensitivity and the specificity fully describe the validity of a screening test.

To appreciate the meaning of the predictive value, PV (the proportion of carcinogens among the substances which are positive to the test) it is necessary to change the frame of reference from that of a screening test to that of a screening programme. By a screening programme we mean the application of a screening test, or a battery of such tests, to a specified group of substances. PV can be interpreted *only* in the context of a screening programme because it simultaneously

TABLE.—*Terms used to describe screening tests and screening programmes*

| Test outcome | Carcinogen* Yes | Carcinogen* No | Total |
|---|---|---|---|
| Positive | a | b | a+b |
| Negative | c | d | c+d |
| Total | a+c | b+d | N=a+b+c+d |

| Term | Definition† |
|---|---|
| Sensitivity | a/(a+c) |
| Specificity | d/(b+d) |
| Predictive value | a/(a+b) |
| Prevalence | (a+c)/N |
| False positive proportion ("rate") | b/(b+d)‡ |
| False negative proportion ("rate") | c/(a+c)‡ |

* As defined by selected criteria.

† Usually each term is multiplied by 100 and expressed as a percentage.

‡ Sometimes wrongly taken as b/(a+b) and c/(c+d) respectively.

reflects 3 things: the sensitivity and specificity of the test being used and the prevalence (proportion) of carcinogens among the substances tested. As the prevalence changes so does the PV of a programme, even though the sensitivity and specificity of the test are constant.

The dependence of the PV on the prevalence can be illustrated by an example based on a widely used screening test (McCann & Ames, 1976). The test correctly classified 157/175 substances considered to be carcinogens and 94/108 non-carcinogens; that is, the test had a sensitivity of 90% and a specificity of 87%. The prevalence of carcinogens among this group of substances was 62% (175/(175+108)) and, under this condition the PV is 92%. However, assuming a constant sensitivity and specificity, if the prevalence (proportion) of carcinogens among the compounds tested had been only 10%, the PV would have been 44% and, if the prevalence had been only 1%, the PV would have been only 7%.

The relationship between prevalence and PV is strong (Figure). Even a highly specific and sensitive test, A, is associated with a PV of less than 90%, until the prevalence reaches 33%. And Test C, with a low specificity, 80%, will be associated with a PV of less than 90% until the prevalence exceeds 75%. This strong dependence of PV on prevalence suffices to exclude it as a measure of test performance. However, PV has another important limitation. For any prevalence less than about 80%, PV is much more dependent on the test's specificity than on its sensitivity (Figure). This can be seen by contrasting the slight reduction in PV when moving from Test A to Test B (reduced sensitivity, constant specificity) with the major reduction when moving from Test A to Test C (constant sensitivity, reduced specificity). Furthermore, the dependence of PV on the specificity becomes stronger as the prevalence declines. The reason for this is easy to comprehend: as the objects of the screen, carcinogens, become rarer they are outnumbered to an increasing

FIGURE.—Predictive value as a function of prevalence for hypothetical tests.
A. (– – – – –) Sensitivity: 95%, Specificity: 95%.
B. (———) Sensitivity: 80%, Specificity: 95%.
C. (–·–·–) Sensitivity: 95% Specificity: 80%.

extent by the many non-carcinogens falsely classified as positive by a non-specific test. This high dependence of PV on specificity and its relative stability in the presence of varying sensitivity should be viewed as a serious limitation, especially in a carcinogen screening test. For most applications such a test should have a high sensitivity, since the penalty associated with "missing" a carcinogen is likely to be much higher than that associated with misclassifying a non-carcinogen.

If PV is not a useful measure of the validity of screening tests, how should such tests be described? Several indices for rating tests have been proposed, of which Youden's J index is probably the best known (Youden, 1950). It is the sum of the sensitivity and the specificity, minus unity. Youden pointed to several strengths of his index, but it has been criticized on several grounds (Greenhouse & Cornfield, 1950). It was pointed out that the sensitivity and specificity of any test will change (in inverse directions) as the criterion of a "positive" test outcome changes. That is, as the criterion of positivity is made more stringent, sensitivity is reduced (some previously detectable carcinogens will be missed) but specificity is increased (a non-carcinogen is less likely to appear positive). Similarly, if the criterion of positivity is relaxed, sensitivity is improved but specificity is reduced. In either case, the index may remain unchanged, rise or fall, depending on the extent of change in each of the 2 basic measures. It was also pointed out that the J index is influenced equally by sensitivity and specificity, a property generally considered undesirable. (This limitation could be overcome readily by some type of weighting procedure.) To these limitations of the J index we would add a third, namely, that any single index of test validity necessarily suppresses valuable information on sensitivity and specificity.

Our suggestion then, is simply that the validation of a screening test be described by presenting the 2 basic measures, sensitivity and specificity. As there are only 2, there seems little purpose in developing a single index which must be limited, to a greater or lesser extent, by one or more of the 3 difficulties mentioned.

While sensitivity and specificity fully describe the validity of a test, 2 additional items of information are necessary for their meaningful interpretation.

The first is the identity of the substances used to evaluate the test. This is important since, for most tests, there is no necessary reason why the same performance should be expected for substances of different classes. There is in fact evidence (Purchase *et al.*, 1978) that tests may exhibit different sensitivity and specificity when applied to different classes of substances, *e.g.* polycyclic hydrocarbons, alkylating agents or aromatic amines.

The second is the criterion of a positive test outcome since this, as already mentioned, directly influences both sensitivity and specificity. When using any one test an effort should be made to select the optimum criterion of a positive outcome, based on an assessment of the relative penalties associated either with missing a carcinogen or with erroneously implicating a non-carcinogen as carcinogenic. Once this criterion is established, improvement can come about only by developing a better test. Another alternative is to use 2 or more tests together or in sequence. The first option, test development, is a continuing process. The second is immediately available and should always be considered.

### REFERENCES

GREENHOUSE, S. W. & CORNFIELD, J., with comment by HOMBURGER, F. (1950) The Youden index. *Cancer*, **3**, 1097.

HENDERSON, M. (1976) Validity of screening. *Cancer*, **37**, 573.

HOLLAND, W. W. (1974) Taking Stock. *Lancet* ii, 1494.

MACMAHON, B. & PUGH, T. F. (1970) *Epidemiology, Principles and Methods.* Boston: Little, Brown and Co. p. 261.

MCCANN, J. & AMES, B. N. (1976) Detection of carcinogens as mutagens in *Salmonella*/microsome test: assay of 300 chemicals: discussion. *Proc. Natl Acad. Sci. U.S.A.*, **73**, 950.

PURCHASE, I. F. H., LONGSTAFF, E., ASHBY, J. & 4 others (1978) An evaluation of six short-term tests for detecting organic chemical carcinogens. *Br. J. Cancer*, **37**, 873.

VECCHIO, T.J. (1966) Predictive value of a single diagnostic test in unselected populations. *New Engl. J. Med.*, **274**, 1171.

YOUDEN, W. J. (1950) Index for rating diagnostic tests. *Cancer*, **3**, 32.


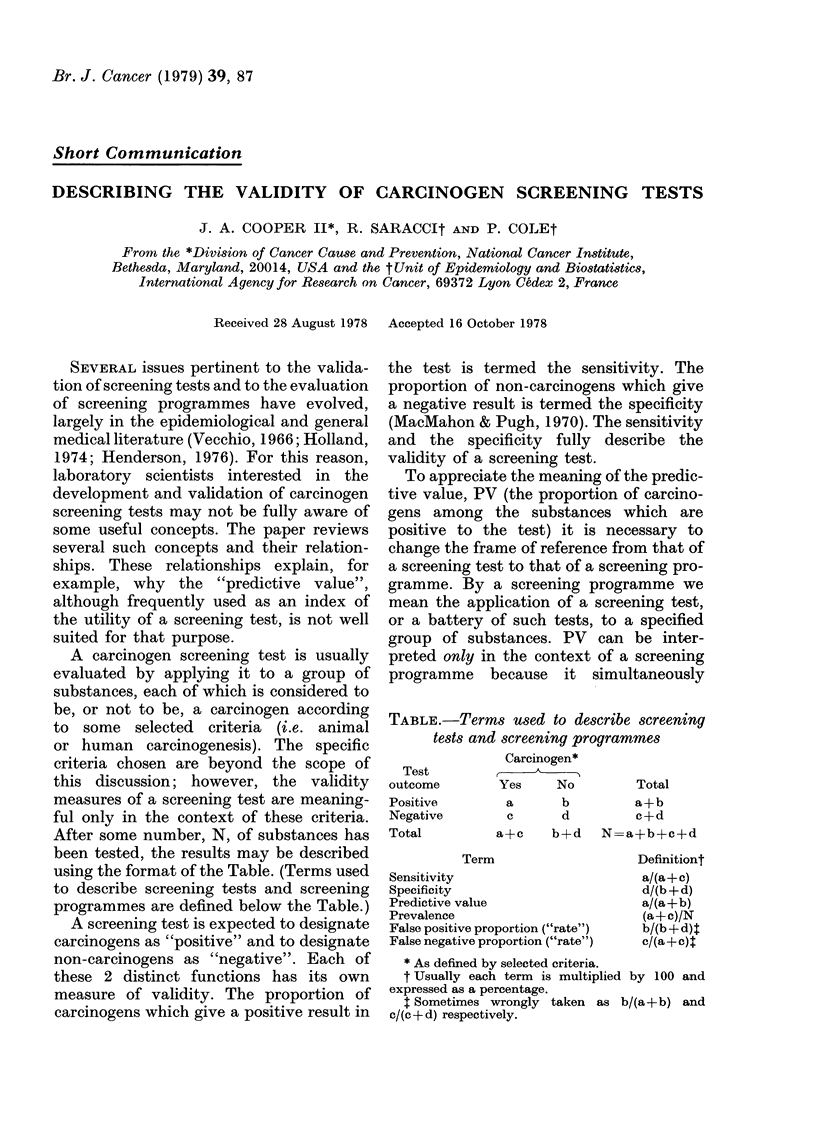

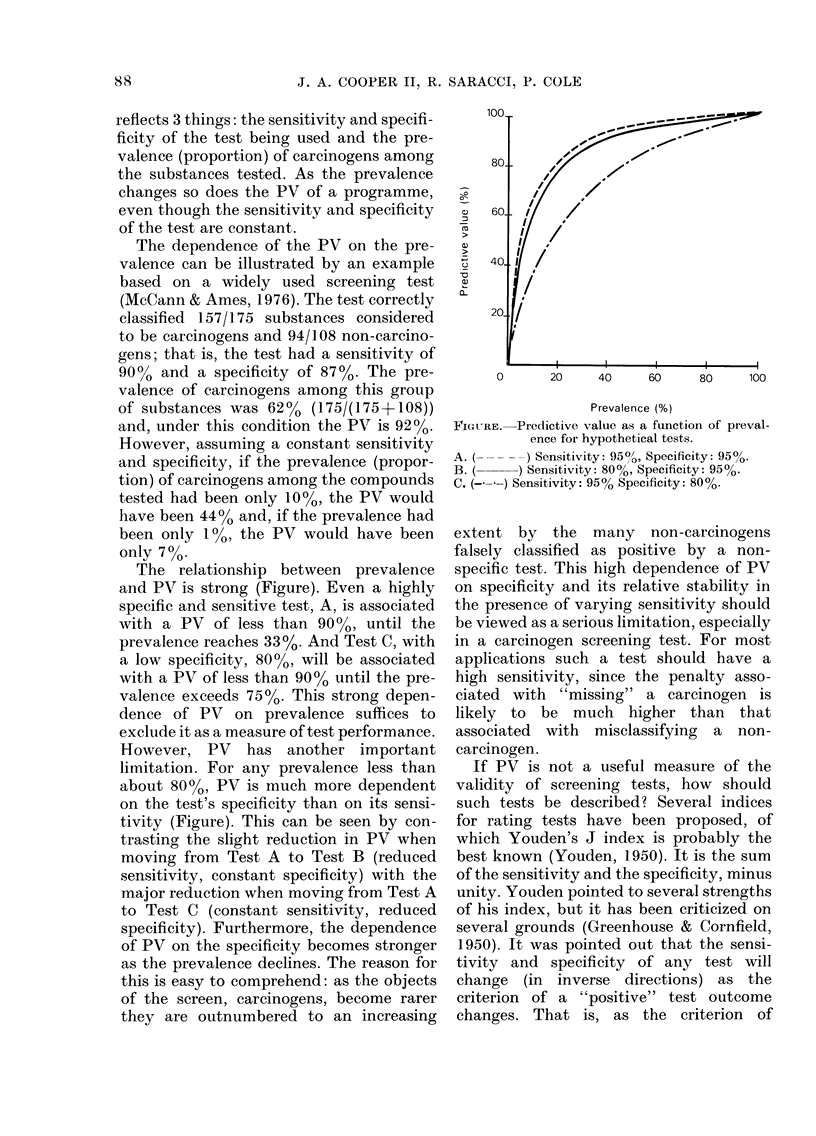

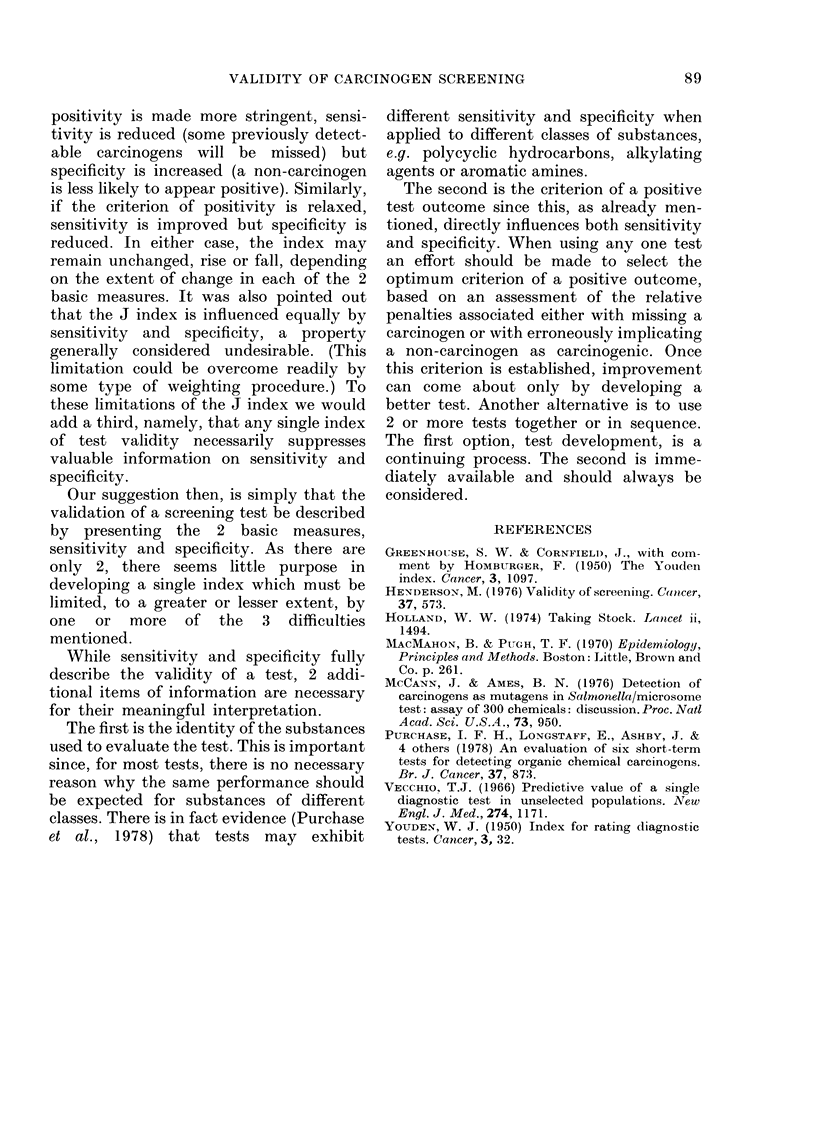


## References

[OCR_00305] GREENHOUSE S. W., CORNFIELD J., HOMBURGER F. (1950). The Youden index: letters to the editor.. Cancer.

[OCR_00308] Henderson M. (1976). Validity of screening.. Cancer.

[OCR_00312] Holland W. W. (1974). Taking stock.. Lancet.

[OCR_00321] McCann J., Ames B. N. (1976). Detection of carcinogens as mutagens in the Salmonella/microsome test: assay of 300 chemicals: discussion.. Proc Natl Acad Sci U S A.

[OCR_00327] Purchase I. F., Longstaff E., Ashby J., Styles J. A., Anderson D., Lefevre P. A., Westwood F. R. (1978). An evaluation of 6 short-term tests for detecting organic chemical carcinogens.. Br J Cancer.

[OCR_00338] YOUDEN W. J. (1950). Index for rating diagnostic tests.. Cancer.

